# Age-Related Differences in Molecular Profiles for Immune Checkpoint Blockade Therapy

**DOI:** 10.3389/fimmu.2021.657575

**Published:** 2021-04-15

**Authors:** Qi-jie Zhang, Jiao-chen Luan, Le-bin Song, Rong Cong, Cheng-jian Ji, Xiang Zhou, Jia-dong Xia, Ning-hong Song

**Affiliations:** ^1^ Department of Urology, The First Affiliated Hospital of Nanjing Medical University, Nanjing, China; ^2^ Department of Dermatology, The First Affiliated Hospital of Nanjing Medical University, Nanjing, China; ^3^ Department of Urology, The Affiliated Kezhou People’s Hospital of Nanjing Medical University, Xinjiang, China

**Keywords:** immune checkpoint blockade, age, immune profiles, therapy efficacy, immunotherapy

## Abstract

Immune checkpoint blockade (ICB) therapies have significantly improved the prognosis and shown considerable promise for cancer therapy; however, differences in ICB treatment efficacy between the elderly and young are unknown. We analyzed the studies enrolled in the meta-analysis using the deft approach, and found no difference in efficacy except melanoma patients receiving anti–PD-1 therapy. Similarly, higher treatment response rate and more favorable prognosis were observed in elderly patients in some cancer types (e.g., melanoma) with data from published ICB treatment clinical trials. In addition, we comprehensively compared immunotherapy-related molecular profiles between elderly and young patients from public trials and The Cancer Genome Atlas (TCGA), and validated these findings in several independent datasets. We discovered a divergent age-biased immune profiling, including the properties of tumors (e.g., tumor mutation load) and immune features (e.g., immune cells), in a pancancer setting across 27 cancer types. We believe that ICB treatment efficacy might vary depending on specific cancer types and be determined by both the tumor internal features and external immune microenvironment. Considering the high mutational properties in elderly patients in many cancer types, modulating immune function could be beneficial to immunotherapy in the elderly, which requires further investigation.

## Introduction

Immune checkpoint blockade (ICB) therapies that enhance antitumor activity of T lymphocytes through blocking immune checkpoints (e.g., PD-1/PD-L1, CTLA-4) have been proven to dramatically improve patient survival in multiple cancer types (1, 2). In most cases, the incidence and mortality of malignancies are associated with increasing age and hence called aging diseases (3). Elderly patients are the major population that requires ICB treatments. However, the immune system changes with age, characterized by altered immune cells and decreased adaptive immunity (4, 5). This process, known as “immunosenescence”, might potentially affect immune responses, but the exact role in ICB immunotherapy remains unknown. Although a few large-scale meta-analyses have been conducted (6–8), debate continues about whether there are differences in immunotherapy efficacy between the elderly and young. Wu et al. claimed that elderly patients receiving ICB treatment had better efficacy than young patients based on a meta-analysis of random clinical trials (6), while two other studies detected no differences in efficacy (7, 8). The underlying cause of the conflict is that age-dependent changes in intratumoral immune populations and response to immunotherapy in the tumor microenvironment remains unclear (9).

Many previous studies have sought to identify biomarkers to predict immunotherapy response. Tumor mutation burden (TMB) is positively correlated with tumor neoantigen load, and high TMB tends to increase the ability of T cells to recognize and kill tumor cells (10, 11). Several clinical trials have demonstrated the relationship between high TMB and ICB benefit (12, 13). PD-L1 is expressed on various cell types, including tumor cells and immune cells, and its expression can help to generate an immunosuppressive tumor microenvironment (14–16). Patients with PD-L1 overexpression are more likely to respond to anti–PD-L1 treatment. Cytolytic activity (CYT) correlates with neoantigen load and amplifications in regions containing immunosuppressive genes, and has predictive value for immunotherapy (17). A T cell-inflamed gene expression profile (GEP) including IFN-γ-responsive genes related to antigen presentation, cytotoxic activity, chemokine expression, and adaptive immune resistance can serve as an independent predictor of response to anti–PD-1 therapy (18–22). Additionally, there are some reports on other biomarkers (23, 24), including protein or mRNA expression of immune checkpoints (e.g., PD-1, CTLA-4), immune cells (e.g., CD4+/CD8+ T cell), aneuploidy score, and BCR/TCR richness. In this study, we comprehensively analyzed age-associated differences in these immune-related molecular biomarkers to better understand age effects on ICB treatment efficacy.

## Materials and Methods

### Effects of Age on ICB Treatment in Clinical Trials

We systematically searched PubMed, Embase, MEDINE, and Web of Science for eligible clinical trials, published before Mar 1st, 2021. The search terms included “PD-1,” “PD-L1,” “CTLA-4,” “immune checkpoint inhibitor,” “atezolizumab,” “avelumab,” “durvalumab,” “ipilimumab,” “nivolumab,” “pembrolizumab,” and “tremelimumab.” Two reviewers independently performed initial research by screening titles and abstracts of retrieved articles. Trials enrolled in our study should meet the following criteria (1): randomized controlled trials concerning cancer therapy (2); participants treated with PD-1 inhibitors, PD-L1 inhibitors, CTLA-4 inhibitors, or their combination compared to placebo or other anti-cancer drugs (3); trials with the hazard ratio (HR) of overall survival (OS) by using 65 as a cut-off age. After integrating our searching results with 2 published meta-analysis studies (6, 8), a total of 34 clinical trials about ICB treatment in 9 different cancer types (16 trials in NSCLC; 6 trials in melanoma; 3 trials in colorectal cancer; 3 trials in GEJC; 2 trials in RCC; 1 trial in SCLC, urothelial, TNBC and mesothelioma) were included in our study (see **Supplementary Table S1** for full details). The deft approach (25) was used for assessment of age effect on the immunotherapy efficacy within each trail, and fixed-effect model (FEM) meta-analysis was applied to combine these estimates among trials. These interactions represent immunotherapy efficacy differences, in which hazard ratio (HR) >1 and HR <1 indicate OS advantage in the young and elderly, respectively.

### Analysis of Molecular Profile in Patients With ICB Treatment

We comprehensively analyzed molecular profile in ICB treatment-related studies, including melanoma, lung cancer, bladder cancer, renal cell carcinoma, and other cancer types (see **Supplementary Table S2** for full details). The profiling comprises of some biomarkers reported in these studies that are potential to alter treatment responsiveness, such as TMB, gene mutation (BRCA2, BRAF, PBRM1), neoantigen, CYT, GEP, gene expression of immune checkpoints (PD-1, PD-L1, PD-L2, CTLA-4), and protein expression of PD-L1. For survival analysis, HR and 95% CI were calculated using Cox proportional hazards model, and Kaplan-Meier survival curve was generated. Fisher’s exact test was used for comparison of individual gene mutation and benefit percentage between the elderly and young, and two-sided Mann-Whitney-Wilcoxon (MWW) test for other molecular features.

### Analysis of Molecular Profile in Patients From The Cancer Genome Atlas (TCGA)

We performed a comprehensive analysis of molecular profiling across 27 cancer types in TCGA with ≥ 20 patients in both the elderly and young groups (see **Supplementary Table S3** for full details). Data of mutation, gene expression, and protein expression were accessible from TCGA (https://portal.gdc.cancer.gov/). Immune checkpoint genes with known co-stimulatory or co-inhibitory effects were summarized by Auslander et al. (26). Six immune c ell populations were evaluated based on Tumor IMmune Estimation Resource (TIMER) (27) (http://cistrome.dfci.harvard.edu/TIMER/). T cell-inflamed gene expression profile (GEP) was calculated according to the signature from Ayers et al. (19). Immune cytolytic activity (CYT) was defined as the geometric mean of GZMA and PRF1 expression (17). Aneuploidy score, T cell receptor/B cell receptor (TCR/BCR) richness, and neoantigen load were derived from Thorsson et al. (https://gdc.cancer.gov/about-data/publications/panimmune) (28). To control for possible confounding factors, including gender (categorical), race (categorical), histologic type (categorical), pathologic stage (categorical), tumor purity (continuous), and smoking history (categorical), between the elderly and young, propensity score (PS) matching analysis was performed (29). Patients in the elderly and young group were matched for 6 factors using PS calculated with the nearest neighbor method. The covariate balance was then checked to assess the adequacy of the propensity model. After balanced, we compared the molecular profile between the elderly and young. P value < 0.05 was considered statistically significant.

### Analysis of Other Data Sets for Independent Verification

To verify above age-biased differences, we obtained TMB in three liver cancer projects (LICA-FR, LINC-JP, LIRI-JP) and one renal cell cancer project (RECA-EU) from the International Cancer Genome Consortium (ICGC) (https://dcc.icgc.org/). Two independent datasets containing gene expression data of lung cancer (GSE19804) (30) and endometrial cancer (GSE17025) (31) were analyzed as well. We performed PS matching analysis as described above and considered P < 0.05 as significance.

## Results

### Pooled Estimate of Overall Survival (OS) From Meta-Analysis

**Figure 1:**
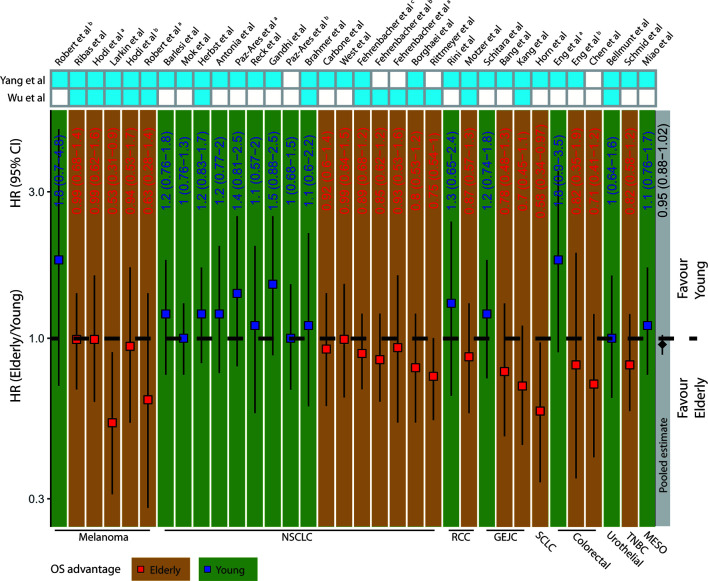
Clinical outcomes in elderly and young patients receiving ICB treatment. The squares and vertical lines represent trial-specific HRs and 95% CIs. Red squares and orange background color indicate OS advantage in elderly patients and blue squares and green background color in young patients. The diamonds show the pooled estimate from fixed-effect meta-analysis. NSCLC, non-small cell lung cancer; RCC, renal cell carcinoma; GEJC, gastric or gastroesophageal junction carcinoma; SCLC, small cell lung cancer; HNC, head and neck cancer; TNBC, triple-negative breast cancer; MESO, mesothelioma.

To determine whether different ICB treatment efficacy exists due to age, we extracted and summarized survival data of the elderly and young from 34 clinical trials (**Supplementary Table S1** **,** **Supplementary Figure S1**). The deft approach was used to calculate HRs specific to each trial, and fixed-effect model for the pooled HRs. We found no significant difference concerning OS (HR=0.95; 95% confidence interval (CI), 0.88–1.02; p= 0.504, **Figure 1**) after pooling the 34 trials. Among 6 melanoma clinical trials, 5 of them showed OS advantage in elderly patients. Interestingly, we observed a particularly clear inconsistency of benefit in NSCLC patients that 9 out of 16 trials showed OS advantage in young patients, while 7 other trials displayed OS advantage in elderly patients. It indicated that merely pooling different trials may not yield a convincing result. Moreover, we performed subgroup analyses according to the types of cancer, ICB treatment, and control choice. We observed insignificant pooled HR for ICB treatment vs. docetaxel (0.92; 95% CI, 0.80–1.05; p= 0.202, **Supplementary Figure S2A**) and ICB treatment vs. placebo (0.98; 95% CI, 0.84–1.15; p= 0.529, **Supplementary Figure S2B**). Similarly, the pooled HR for anti–CTLA-4 trials (1.04; 95% CI, 0.84–1.29; p= 0.219, **Supplementary Figure S2C**) remained insignificant, whereas anti–PD-1/PD-L1 trials (0.94; 95% CI, 0.86–1.02; p= 0.098, **Supplementary Figure S2D**) showed a trend toward better OS in the elderly. Moreover, different from anti–CTLA-4 trials (1.02; 95% CI, 0.8–1.31; p= 0.942, **Supplementary Figure S2E**), the pooled HR for anti–PD-1 trials (0.559; 95% CI, 0.358–0.871; p= 0.010, **Supplementary Figure S2F**) showed significant OS advantage in elderly patients among the melanoma subgroup.

### Age-Bias of Potential Molecular Biomarkers in ICB Treatment

**Figure 2:**
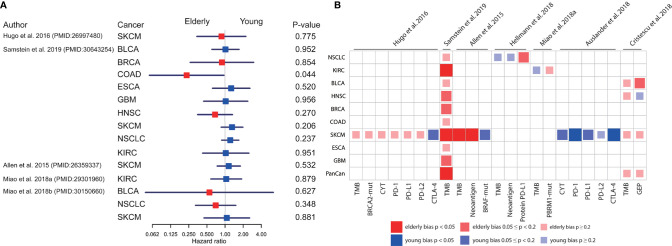
Comparison of overall survival and molecular profiles in elderly and young patients with ICB treatments. **(A)** Univariate analyses based on Cox proportional hazards model for elderly and young patients receiving ICB treatment in 9 cancer types from 5 datasets. The squares and horizonal lines represent trial-specific HRs and 95% CIs. Square colors represent OS advantage in elderly (red) and young (blue) patients. **(B)** The age-bias of molecular biomarkers reported in patients with ICB treatment across multiple cancer types from 7 datasets. Fisher’s exact test for individual gene mutation, and two-sided Mann-Whitney-Wilcoxon test for other biomarkers. TMB, tumor mutation burden; CYT, cytolytic activity; GEP, T cell-inflamed gene expression profile; BLCA, bladder urothelial carcinoma; BRCA, breast invasive carcinoma; COAD, colon adenocarcinoma; ESCA, esophageal carcinoma; GBM, glioblastoma; HNSC, head and neck squamous cell carcinoma; KIRC, kidney renal clear cell carcinoma; SKCM, skin cutaneous melanoma; NSCLC, non-small cell lung cancer.

**Figure 3:**
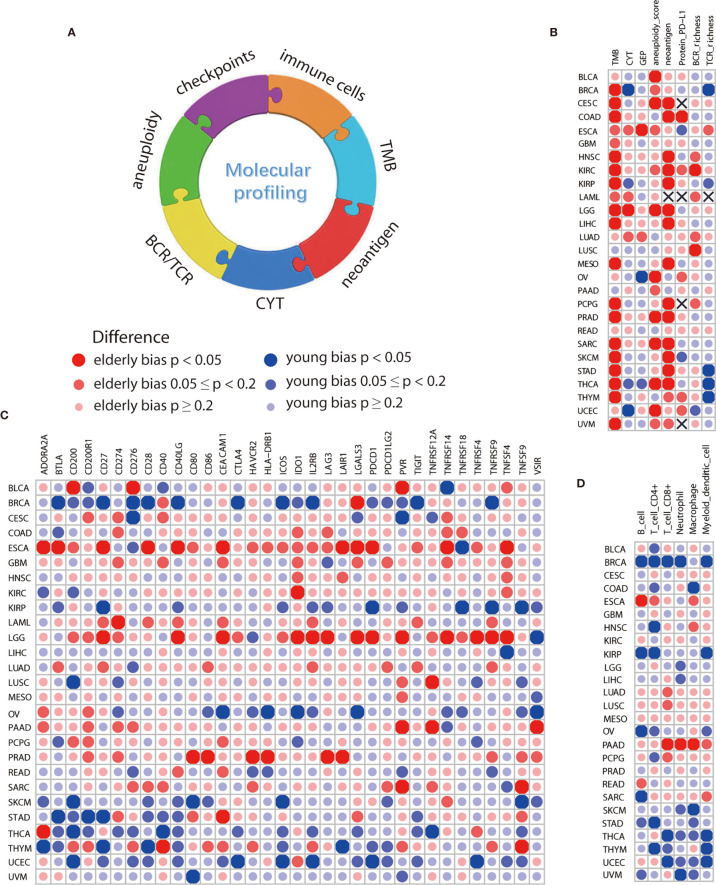
Comparison of immune features between elderly and young patients from TCGA. **(A)** Overview of age-associated immune features comprising TMB, CYT, neoantigen, aneuploidy, BCR/TCR richness, immune cells, and checkpoints. **(B)** Differences in molecular biomarkers reported in immunotherapy-related studies, including TMB, CYT, GEP, neoantigen and protein PD-L1, and some potential biomarkers, including aneuploidy, BCR and TCR richness. **(C)** Differences in mRNA expression level of 34 immune checkpoints, including both stimulatory and inhibitory immune checkpoints. **(D)** Differences in 6 types of immune cells, including B cell, CD4+ T cell, CD8+ T cell, neutrophil, macrophage, and myeloid dendritic cell. Propensity score with the nearest neighbor method was used for comparison of immune features between the elderly and young. Sample size for each cancer type was displayed in **Supplementary Table S3**.

To explore the possible mechanism underlying age-associated immunotherapy responsiveness, 7 eligible ICB treatment datasets with molecular profile for each patient were analyzed after screening (18, 26, 32–36). We found a diverse survival pattern concerning OS between the elderly and young patients with ICB treatment (**Figure 2A**). Elderly patients with colorectal cancer (COAD) had better OS than young patients (p=0.044)(**Supplementary Figure S3A**). Compared with young patients, OS tended to be better in elderly patients with HNSC and NSCLC. In addition, we observed a significantly higher response rate in elderly melanoma patients from 3 studies (26% vs. 9%, p=0.019; 27% vs. 8%, p=0.012; 64% vs. 32%, p=0.037) (**Supplementary Figures S3B–D** **)**. As regards molecular markers (**Figures 2B, 3A**), tumor mutation burden (TMB) was significantly higher in elderly patients with melanoma (p<0.001, p=0.015, **Supplementary Figures S3E, F**), clear cell renal cell carcinoma (KIRC)(p=0.044, **Supplementary Figure S3G**), and pan-cancer (p<0.001, **Supplementary Figure S3H**) than young patients. Similarly, significantly higher neoantigen load was observed in elderly patients with melanoma (p=0.016, **Supplementary Figure S3I**). In contrast, elderly melanoma patients seemed to have lower CTLA-4 (p=0.001, **Supplementary Figure S3J**) and PD-1 (p=0.033, **Supplementary Figure S3K**) expression than young patients. Other biomarkers showed no significant difference between the elderly and young.

### A Diverse Age-Bias of Immunologic Characteristics in Multiple Cancer Types

No significant difference on confounding factors including gender, race, histologic type, pathologic stage, tumor purity, and smoking history between the elderly and young was observed by using PS matching analysis (**Supplementary Figure S4**). After controlling for these factors, we found that elderly melanoma patients had more TMB (p=0.002, **Figure 3B**, **Supplementary Figure S5A**) and neoantigen load (p=0.011, **Figure 3B** and **Supplementary Figure S5B**), which was consistent with immunotherapy data sets. Similarly, several other cancer types showed elderly-bias towards TMB, neoantigen load, and aneuploidy score (**Figure 3B** and **Supplementary Figures S5C–F** **)**. In addition, elderly patients exhibited higher gene expression of immune checkpoints (**Figure 3C**) and higher levels of immune cells (**Figure 3D**) in some cancers, including esophageal carcinoma (ESCA), brain lower grade glioma (LGG), and prostate adenocarcinoma (PRAD). Protein PD-L1 significantly elevated in elderly patients in COAD (p=0.028, **Figure 3B**, **Supplementary Figure S5G**). By contrast, young-biased immune traits appeared in several cancers as well. For instance, a few biomarkers significantly elevated in young patients with breast invasive carcinoma (BRCA), including CYT (p=0.049, **Figure 3B**, **Supplementary Figure S5H**), TCR richness (p=0.005, **Figure 3B** and **Supplementary Figure S5I**), 9 out of 34 immune checkpoints (**Figure 3C**), and 5 out of 6 immune cells (**Figure 3D**). Interestingly, young-bias existed in both stimulatory (CD27, ICOS, and so on) and inhibitory (BTLA, CTLA-4, and so on) immune checkpoints. Except macrophage, other five types of cells (B cell, CD4+ T cell, CD8+ T cell, neutrophil, and myeloid dendritic cell) showed young-bias.

### Verification of Age-Bias Using Independent Data Sets

**Figure 4:**
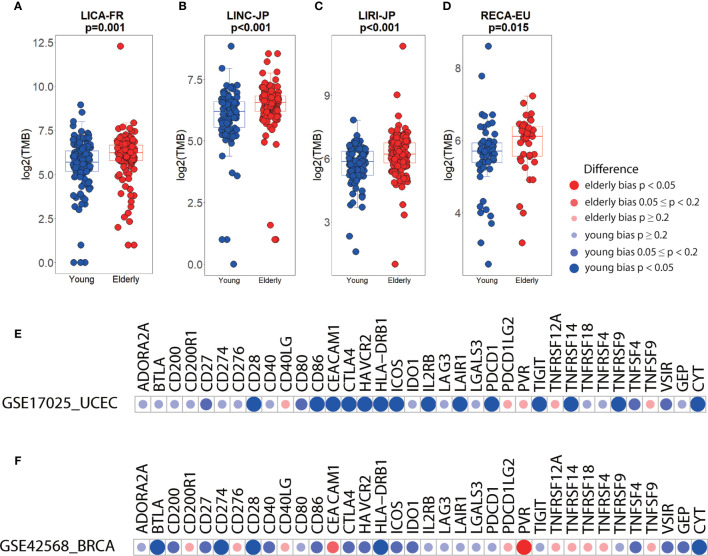
Differences in immune biomarkers between elderly and young patients in independent datasets. **(A–D)** Boxplots showing the differences in TMB between elderly and young patients with liver cancer (a, LICA-FR; b, LINC-JP; c, LIRI-JP) and renal cell cancer (d, RECA-EU). Boxplot center line, bounds of box and whiskers represent median, inter-quartile range and outliers. **(E)** Checkpoints, GEP, and CYT between elderly and young patients with UCEC (GSE17025, elderly= 26, young= 60) and BRCA (GSE42568, elderly= 36, young= 68).

Examination of several independent datasets was performed for validating the results from TCGA. Consistent with the elderly-bias on TMB in liver hepatocellular carcinoma (LIHC) (**Figure 3B**), significantly higher TMB was observed in three independent datasets (LICA-FR, p=0.001; LINC-JP, p<0.001; LIRI-JP, p<0.001) (**Figures 4A–C**). As for kidney cancer, TMB was significantly elevated in elderly patients with kidney renal clear cell carcinoma (KIRC) and kidney renal papillary cell carcinoma (KIRP) from TCGA (**Figure 3B**) and in elderly patients from another independent dataset (RECA-EU, p=0.015) (**Figure 4D**). Regarding uterine corpus endometrial carcinoma (UCEC), we observed a young-bias pattern concerning immune checkpoints (CTLA-4, ICOS, and so on) and CYT (p=0.013) and validated it in an independent dataset (**Figure 4E** **)**. Similarly, young-biased immune checkpoints (BTLA, CD28, and so on) and CYT in BRCA were observed in TCGA and validated in another independent dataset (**Figure 4F** **)**.

## Discussion

Accumulating evidence indicates that differences in the immune function between the young and elderly are closely associated with the pathogenesis of infections, autoimmune diseases, and malignancies and the response to vaccine, whereas the role in cancer immunotherapy is still ambiguous (37–39). The changes in the immune function of the elderly, also called immunosenescence, refer to a natural process occurring with age and leading to a decline in immune function (40). Due to this, young patients with tumors seem to be more likely to benefit from immunotherapy. However, in this study we compared survival differences between the elderly and young *via* the deft approach in clinical trials from meta-analysis (6, 8), and found no significant differences in most trials. In contrast, elderly patients may have a significantly better OS in some cancers, especially melanoma. Totally 5 of 6 melanoma clinical trials exhibited OS advantage in the elderly. Additionally, we found that elderly patients with melanoma receiving anti–PD-1 therapy were associated with better OS. In melanoma, the response rate significantly elevated in the elderly compared with young patients based on 3 ICB trials (33, 41, 42). These findings were also consistent with the results reported in other studies (43–45). Overall, these lines of evidence suggest that superior efficacy in the elderly may truly exist in some cancer types. Although a majority of current studies defined the cut-off age as 65 years old, a small part of them took 70 or 75 years old as cut-off value and observed the reduced efficacy in elderly patients (46, 47). This inconsistency may be explained by patients’ own physical conditions, as patients aged over 70 or 75 are more susceptible to other diseases, such as respiratory, cardiovascular and cerebrovascular diseases, which might correlate with reduced OS and affect the immune system to some degree as well.

A few studies have been conducted to investigate potential reasons for superior efficacy in the elderly, which mainly focused on the function of the immune system and immune cells in it (48–51). For example, Kugel et al. believed that fewer Tregs and more CD8+ T cells may be responsible for the better response to anti–PD-1 treatment in the elderly based on an animal and clinical research (45). After analysis of molecular profiling in patients receiving ICB treatment, we found that the elderly melanoma patients had more TMB and neoantigen than the young. However, it may be restricted by some limitations, including small sample size and effects of confounding factors. Therefore, we took the TCGA and other independent datasets as the validation cohorts, performed PS matching analysis to avoid effects of confounding factors, and finally obtained similar results. One other suggestion could be that different intrinsic tumor properties partly contribute to different prognosis and response rate between the elderly and young. After exposure to experimental and intrinsic mutagens for long periods, the formers have more mutations and neoantigens which can be targeted by the host immune system (52). In addition to melanoma, similar results were seen in many other cancer types, such as HNSC, LIHC, COAD, and so on, which further supported the existence of different tumor properties between the elderly and young. It is, however, not applicable to all cancer types, such as LUSC and LUAD, and no obvious age-bias on efficacy was observed in lung cancer patients. Due to limited amount of available clinical trials for analysis, except melanoma and lung cancer, the association between efficacy and different age-related tumor properties is yet unknown and deserves further study.

Certainly, both tumor antigens and a functional immune system are necessary to identify and kill tumor cells accurately and efficiently (53–55). There is a body of evidence that almost all measures of innate and adaptive immunity are different between the elderly and young (5, 49). A remarkable feature is aging-related degeneration of the thymus, where T cells differentiate, develop, and mature. It has been reported that few CD8+ naïve T cells with shrunken antigen receptor repertoire, which participated in the process of antigen recognition (56, 57), was possessed for neoantigen in the elderly, indicating the increased difficulty for the elderly patients to recognize tumor antigens. This is only speculation, however, as no data to date is available to directly confirm the impaired ability of recognizing tumor neoantigens in the elderly. Additionally, stem cell memory T cells (TSCM) can produce more terminally differentiated daughter cells expressing effector molecule, which is crucial for immunotherapy (58, 59); however, no significant effects of age on TSCM were observed throughout life (60, 61). The characteristics of immune system in humans and other species were not exactly the same, and most of the current knowledge about age-related differences in immune function came from animal studies, especially mouse models, which limited their application for clinical immunotherapy to some extent. In our study, a divergent age-biased immune profiling was observed in a pancancer setting across 27 cancer types. Among these cancer types, part of them showed young-biased or elderly-biased immune traits, while a substantial portion of them showed no obvious age-bias. Intriguingly, stimulatory and inhibitory immune checkpoints have essential roles in immune activation and suppression (62), while both of them showed consistent changes with age in some cancer types. Thus far, there is no clear evidence that the functions of ICB-treatment associated immune components are impaired in the elderly patients. We hypothesize that immunotherapy efficacy might be tumor-specific, which is determined by both the tumor itself and the tumor immune microenvironment. Further in-depth investigation is needed to figure out whether and how immune system changed with age, which might help to predict and improve immunotherapy efficacy in the elderly.

A limitation of our study is the common shortcomings of meta-analysis which have been discussed in the research community, such as lack of individual patient data and inconsistent selection criteria. Another limitation concerns incomplete clinical information of patients in some cancer types from public datasets. In spite of these limitations, the study certainly adds to our understanding of differences in immunotherapy efficacy and immune profiling between the elderly and young.

## Conclusions

In conclusion, age-biased immune profiling of the tumor properties and immune features are observed *via* a comprehensive pan-cancer analysis, and the balance between them could be involved in determining the ICB treatment efficacy, which might be beneficial to immunotherapy of elderly patients in the future.

## Data Availability Statement

The original contributions presented in the study are included in the article/**Supplementary Material**. Further inquiries can be directed to the corresponding authors.

## Author Contributions

N-HS and J-DX conceived and designed the study. Q-JZ, J-CL, and L-BS were responsible for data acquisition and analysis. Q-JZ, RC, C-JJ, and XZ wrote and revised the manuscript. All authors contributed to the article and approved the submitted version.

## Funding

This study was funded by Medical key talent of Jiangsu Province (grant ZDRCA2016009); Fellowship of China Postdoctoral Science Foundation (grant 2020M671393); Jiangsu Province Postdoctoral Research Support Project (grant 2020Z134); the National Natural Science Foundation of China (81871151, 81971377).

## Conflict of Interest

The authors declare that the research was conducted in the absence of any commercial or financial relationships that could be construed as a potential conflict of interest.

## Supplementary Material

Supplementary Figure 1 Summary information of overall survival data in our meta-analysis. Hazard ratios (HRs) for overall survival (OS) of elderly and young patients receiving intervention (immune checkpoint blockage, ICB) and control treatments in 39 clinical trials from Wu et al. and Yang et al. Cells filled with ligfht blue mean the study included in this meta-analysis. Red dots indicate trial-specific HRs in elderly patents and blue dots in young patients. The vertical lines represent 95% CIs. OS advantage in elderly and young patients are denoted by orange and green background color. NSCLC, non-small cell lung cancer; RCC, renal cell carcinoma; GEJC, gastric or gastroesophageal junction carcinoma; SCLC, small cell lung cancer; HNC, head and neck cancer; TNBC, triple-negative breast cancer; MESO, mesothelioma.

Supplementary Figure 2 Clinical outcomes between elderly and young patients with ICB treatments. **(A, B)** The correlation of treatment outcome and age based on different control arms, docetaxel **(A)** and placebo **(B)**.** (C, D)** The correlation of treatment outcome and age based on different ICB treatment types, anti-CTLA-4 **(C)** and anti-PD-1/PD-L1 **(D)**. **(E, F)** The correlation of treatment outcome and age in melanoma patients receiving anti-CTLA-4 **(E)** and anti-PD-1 **(F)** therapy. The squares and vertical lines represent trial-specific HRs and 95% CIs. Red squares and orange background color indicate OS advantage in elderly patients and blue squares and green background color in young patients. The diamonds show the pooled estimate from fixed-effect meta-analysis. NSCLC, non-small cell lung cancer; RCC, renal cell carcinoma; GEJC, gastric or gastroesophageal junction carcinoma; SCLC, small cell lung cancer; HNC, head and neck cancer; TNBC, triple-negative breast cancer; MESO, mesothelioma.

Supplementary Figure 3 Comparison of overall survival, response rate, and molecular features between elderly and young patients with ICB treatment. **(A)** Kaplan Meier survival curve of COAD patients receiving anti-PD-1 from one dataset (PMID: 30643254). **(B–D)** Differences in response rate between elderly and young SKCM patients with anti-CTLA-4 therapy from two datasets (PMID: 26359337, 30150660) and anti-PD-1 therapy from one dataset (PMID: 32708981). **(E–H)** Differences in TMB between elderly and young patients with SKCM (PMID: 30643254, 26359337), KIRC (PMID: 30643254), and pancancer (PMID: 30643254). (i-k) Differences in neoantigen (PMID: 26359337), CTLA-4 (PMID: 30127394) and PD-1 (PMID: 30127394) between elderly and young patients with SKCM. TMB, tumor mutation burden; COAD, colon adenocarcinoma; KIRC, kidney renal clear cell carcinoma; SKCM, skin cutaneous melanoma.

Supplementary Figure 4 Potential confounding factors and propensity score between elderly and young patients in 27 cancer types. **(A)** Heatmap shows p values with significance (red dot) or non-significance (grey dot) at a cutoff p-value=0.05 (two-sided Mann-Whitney-Wilcoxon test for tumor purity and Fisher’s exact test for gender, race, histological type, pathologic stage, and smoking). NA, not available. **(B)** Histograms of raw and matched propensity score between elderly and young patients across 27 cancer types.

Supplementary Figure 5 Differences in molecular features between elderly and young patients from TCGA. TMB **(A)** and neoantigen **(B)** in elderly and young patients with SKCM. TMB **(C)**, neoantigen **(D)**, and aneuploidy score **(E)** in elderly and young patients with PRAD. Protein PD-L1 **(G)** expression in elderly and young patients with COAD. TMB **(F)**, CYT **(H)**, and TCR richness **(I)** in elderly patients with BRCA. TMB, tumor mutation burden; CYT, cytolytic activity; GEP, T cell-inflamed gene expression profile; TCR, T cell receptor; BRCA, breast invasive carcinoma; COAD, colon adenocarcinoma; PRAD, prostate adenocarcinoma; SKCM, skin cutaneous melanoma.
