# Supplementary figures and images for: Age-Related Differences in Molecular Profiles for Immune Checkpoint Blockade Therapy
Source: Front Immunol. 2021 Apr 15;12:657575. doi: 10.3389/fimmu.2021.657575 (PMC8082107; doi:10.3389/fimmu.2021.657575)

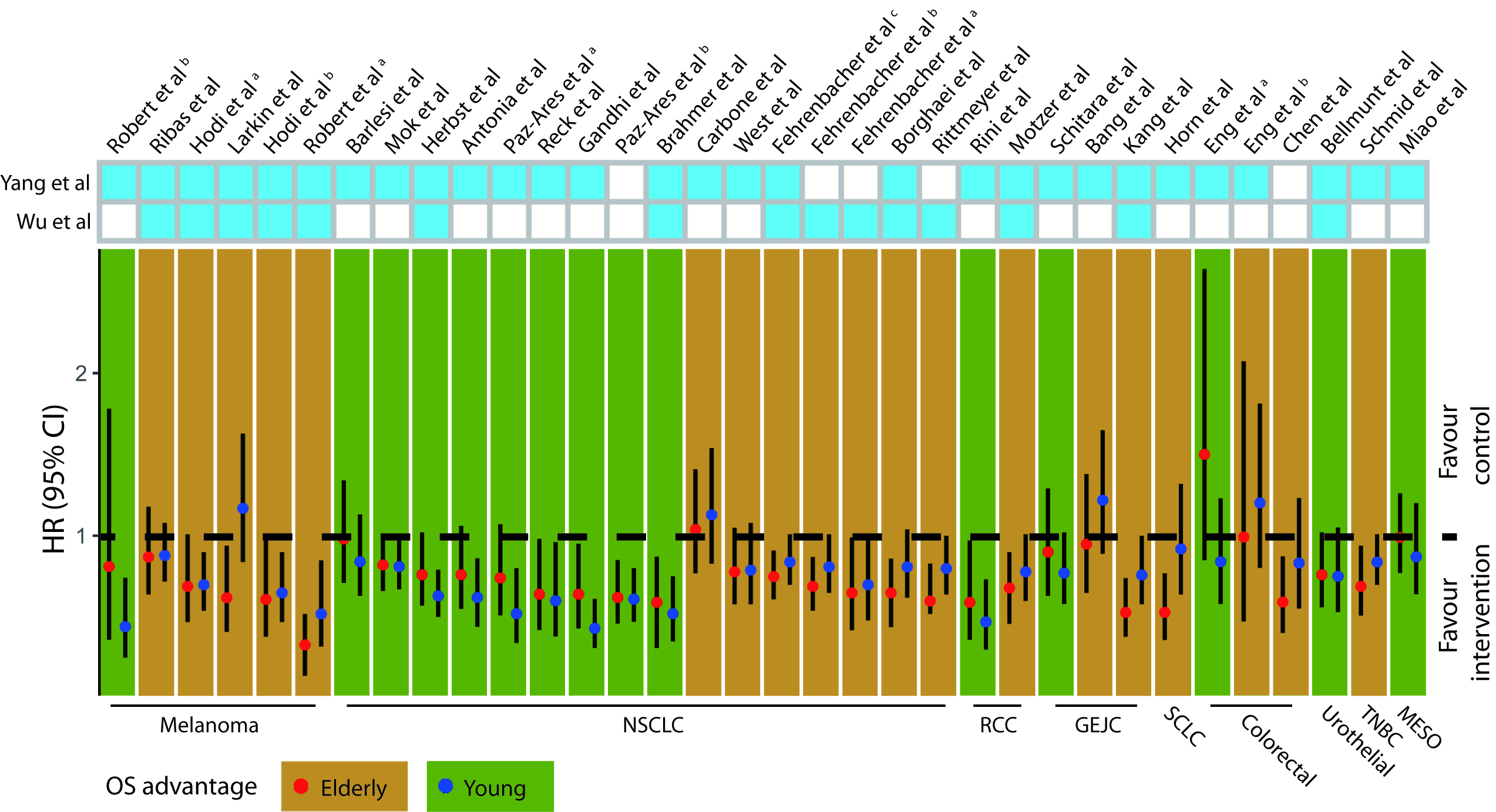

Supplement: Supplementary Figure 1 — Summary information of overall survival data in our meta-analysis. Hazard ratios (HRs) for overall survival (OS) of elderly and young patients receiving intervention (immune checkpoint blockage, ICB) and control treatments in 39 clinical trials from Wu et al. and Yang et al. Cells filled with ligfht blue mean the study included in this meta-analysis. Red dots indicate trial-specific HRs in elderly patents and blue dots in young patients. The vertical lines represent 95% CIs. OS advantage in elderly and young patients are denoted by orange and green background color. NSCLC, non-small cell lung cancer; RCC, renal cell carcinoma; GEJC, gastric or gastroesophageal junction carcinoma; SCLC, small cell lung cancer; HNC, head and neck cancer; TNBC, triple-negative breast cancer; MESO, mesothelioma. [file Image_1.tif]

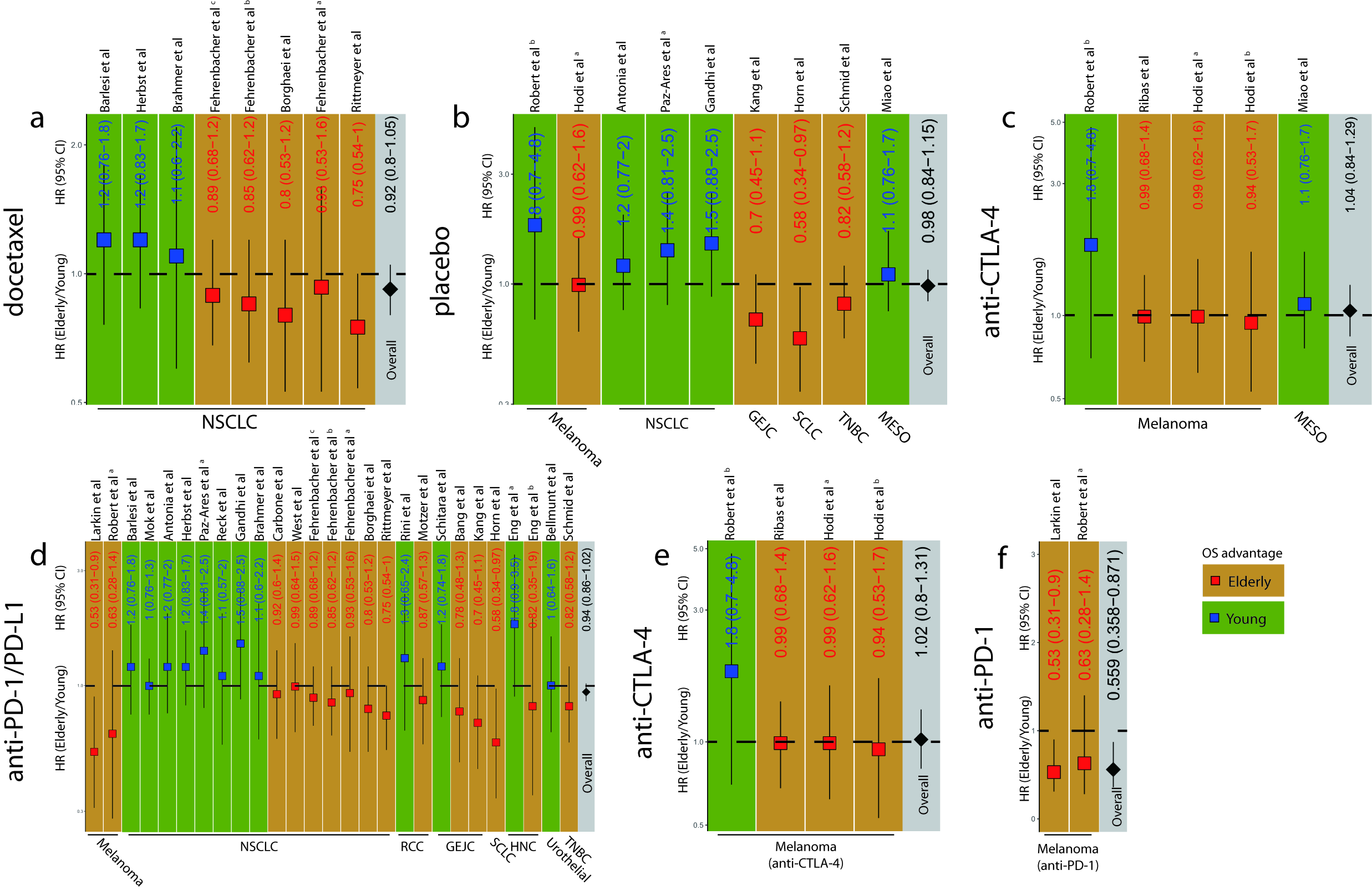

Supplement: Supplementary Figure 2 — Clinical outcomes between elderly and young patients with ICB treatments. (A, B) The correlation of treatment outcome and age based on different control arms, docetaxel (A) and placebo (B). (C, D) The correlation of treatment outcome and age based on different ICB treatment types, anti-CTLA-4 (C) and anti-PD-1/PD-L1 (D). (E, F) The correlation of treatment outcome and age in melanoma patients receiving anti-CTLA-4 (E) and anti-PD-1 (F) therapy. The squares and vertical lines represent trial-specific HRs and 95% CIs. Red squares and orange background color indicate OS advantage in elderly patients and blue squares and green background color in young patients. The diamonds show the pooled estimate from fixed-effect meta-analysis. NSCLC, non-small cell lung cancer; RCC, renal cell carcinoma; GEJC, gastric or gastroesophageal junction carcinoma; SCLC, small cell lung cancer; HNC, head and neck cancer; TNBC, triple-negative breast cancer; MESO, mesothelioma. [file Image_2.tif]

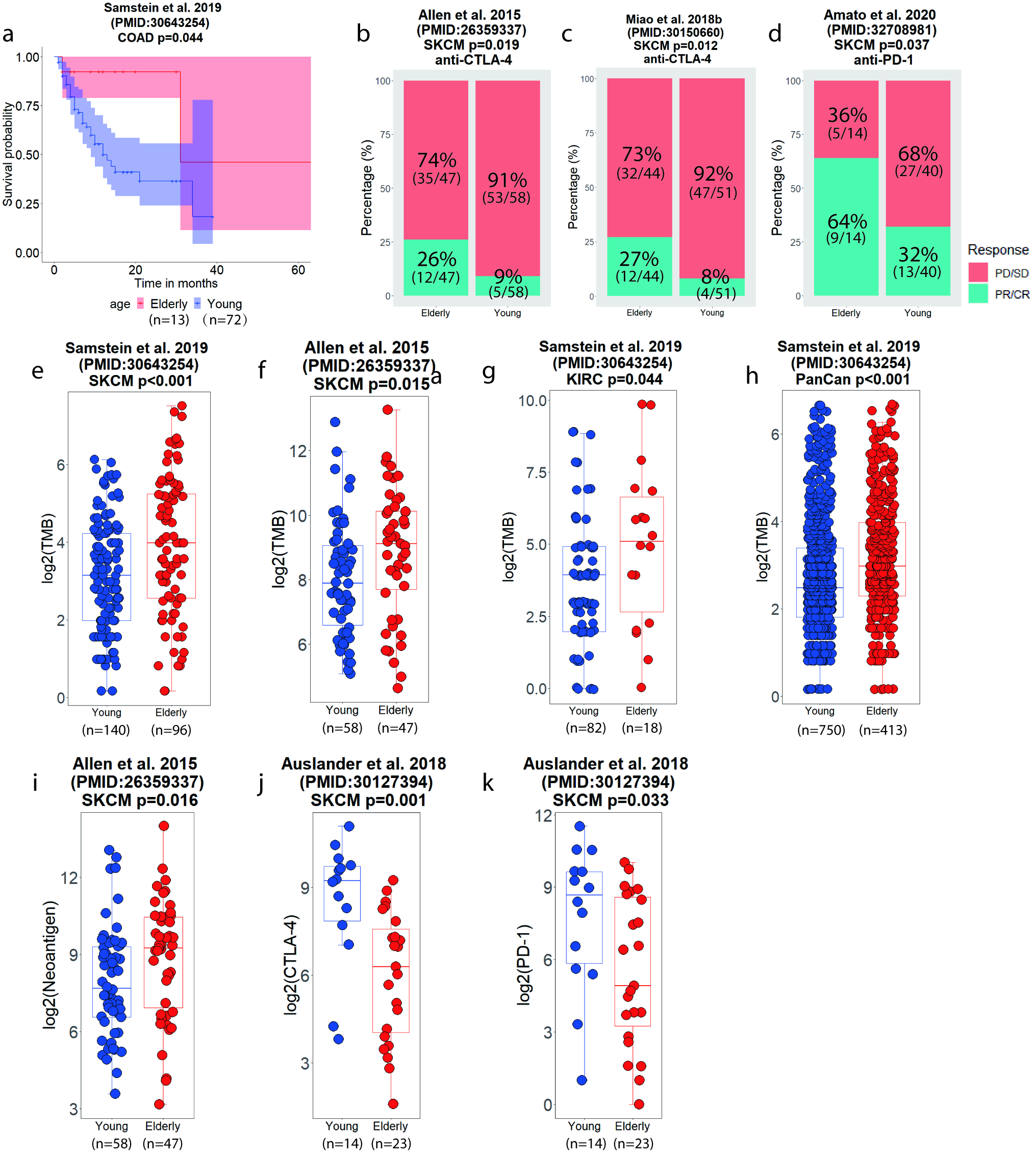

Supplement: Supplementary Figure 3 — Comparison of overall survival, response rate, and molecular features between elderly and young patients with ICB treatment. (A) Kaplan Meier survival curve of COAD patients receiving anti-PD-1 from one dataset (PMID: 30643254). (B–D) Differences in response rate between elderly and young SKCM patients with anti-CTLA-4 therapy from two datasets (PMID: 26359337, 30150660) and anti-PD-1 therapy from one dataset (PMID: 32708981). (E–H) Differences in TMB between elderly and young patients with SKCM (PMID: 30643254, 26359337), KIRC (PMID: 30643254), and pancancer (PMID: 30643254). (i-k) Differences in neoantigen (PMID: 26359337), CTLA-4 (PMID: 30127394) and PD-1 (PMID: 30127394) between elderly and young patients with SKCM. TMB, tumor mutation burden; COAD, colon adenocarcinoma; KIRC, kidney renal clear cell carcinoma; SKCM, skin cutaneous melanoma. [file Image_3.tif]

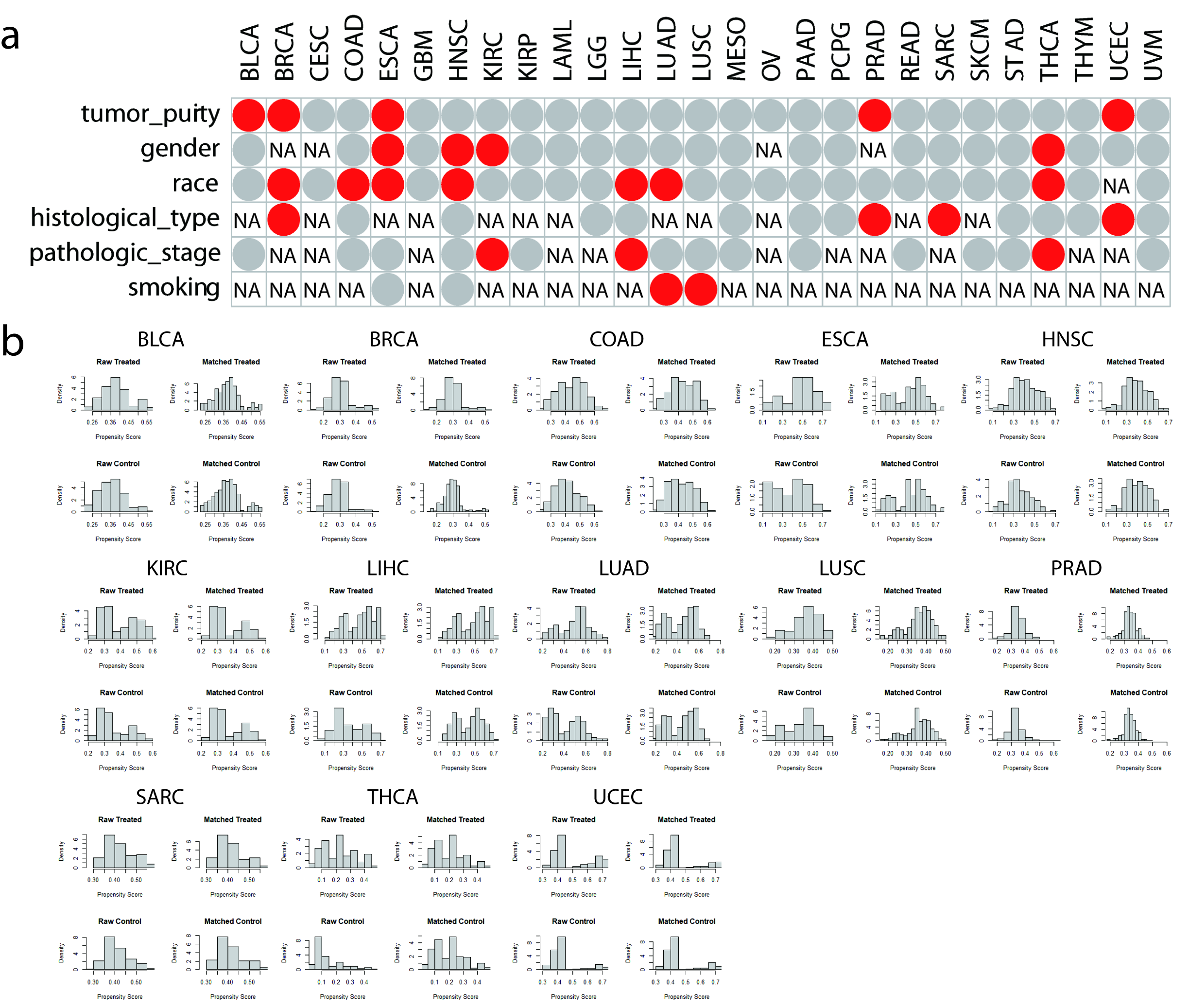

Supplement: Supplementary Figure 4 — Potential confounding factors and propensity score between elderly and young patients in 27 cancer types. (A) Heatmap shows p values with significance (red dot) or non-significance (grey dot) at a cutoff p-value=0.05 (two-sided Mann-Whitney-Wilcoxon test for tumor purity and Fisher’s exact test for gender, race, histological type, pathologic stage, and smoking). NA, not available. (B) Histograms of raw and matched propensity score between elderly and young patients across 27 cancer types. [file Image_4.tif]

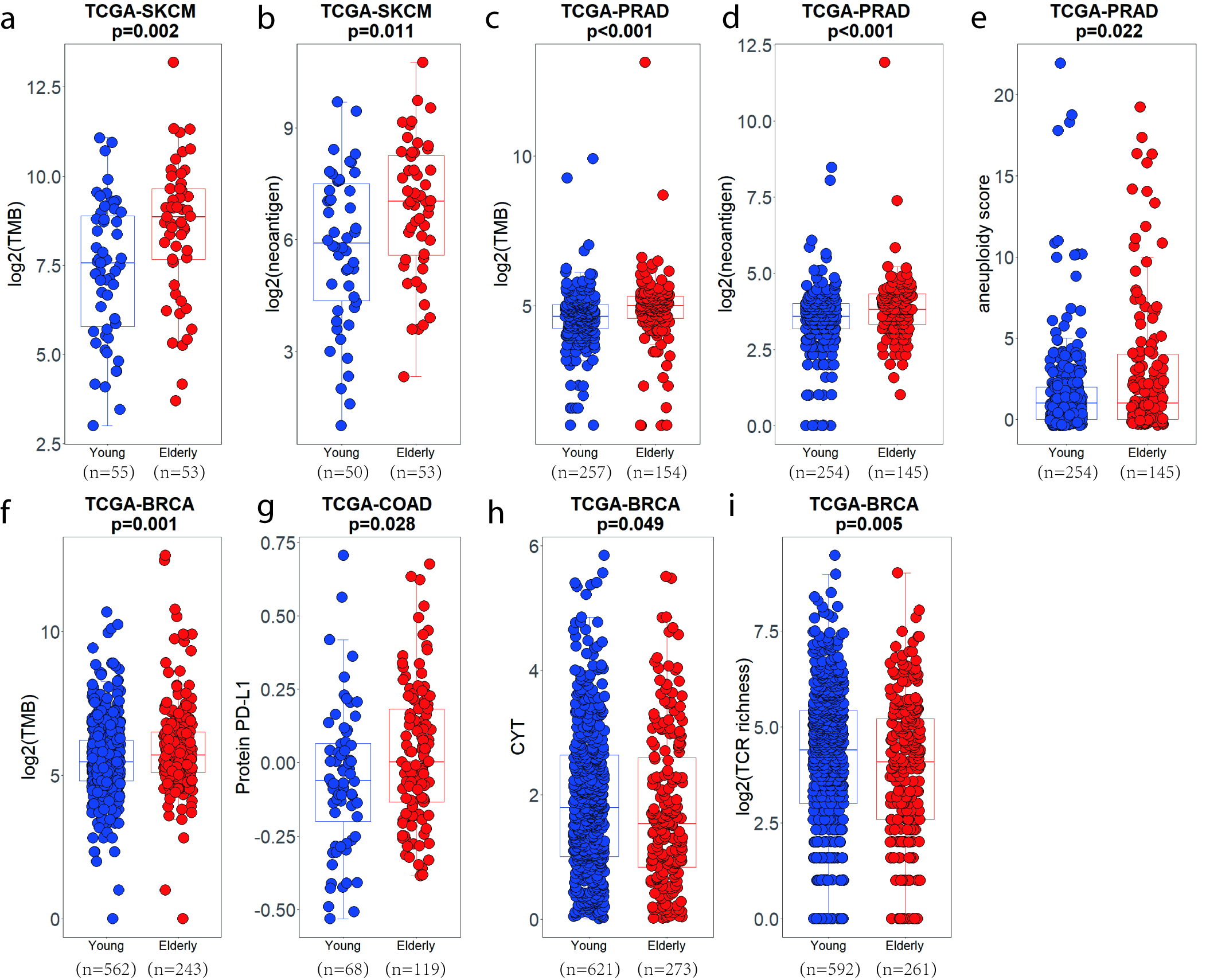

Supplement: Supplementary Figure 5 — Differences in molecular features between elderly and young patients from TCGA. TMB (A) and neoantigen (B) in elderly and young patients with SKCM. TMB (C), neoantigen (D), and aneuploidy score (E) in elderly and young patients with PRAD. Protein PD-L1 (G) expression in elderly and young patients with COAD. TMB (F), CYT (H), and TCR richness (I) in elderly patients with BRCA. TMB, tumor mutation burden; CYT, cytolytic activity; GEP, T cell-inflamed gene expression profile; TCR, T cell receptor; BRCA, breast invasive carcinoma; COAD, colon adenocarcinoma; PRAD, prostate adenocarcinoma; SKCM, skin cutaneous melanoma. [file Image_5.tif]
