# Supplementary material for: Age-Related Differences in Molecular Profiles for Immune Checkpoint Blockade Therapy
Source: Front Immunol. 2021 Apr 15;12:657575. doi: 10.3389/fimmu.2021.657575 (PMC8082107; doi:10.3389/fimmu.2021.657575)
Supplement: Supplementary file 6 [file Table_1.docx]

**Supplementary Table S1.** Main characteristics of the clinical trials with ICB treatment enrolled in our meta-analysis.

| **Dataset**  **(PMID)** | **Author**  **Year** | **Cancer**  **type** | **Intervention**  **(No.)** | **Control Treatment**  **(No.)** | **Age (No.)** | | **Overall Survival**  **HR, 95%CI** | | **Wu et al** | **Yang et al** | **Previous Treatment** |
| --- | --- | --- | --- | --- | --- | --- | --- | --- | --- | --- | --- |
|  |  |  |  |  | **Young**  **(<65)** | **Elderly**  **(≥65)** | **Young**  **(<65)** | **Elderly**  **(≥65)** |  |  |  |
| 30280658 | Antonia et al 2018 | NSCLC | Durvalumab  (473) | Placebo  (236) | 391 | 322 | 0.62  (0.44-0.86) | 0.76  (0.55-1.06) | No | Yes | Chemoradiotherapy |
| 30052729 | Bang et al  2018 | GEJC | Avelumab  (185) | Chemotherapy  (186) | 230 | 141 | 1.22  (0.89-1.65) | 0.95  (0.65-1.38) | No | Yes | Two prior lines of systemic treatment |
| 30262187 | Barlesi et al  2018 | NSCLC | Avelumab  (264) | Docetaxel  (265) | 279 | 250 | 0.84  (0.63-1.13) | 0.98  (0.71-1.34) | No | Yes | Platinum-based chemotherapy |
| 28212060 | Bellmunt et al 2017 | Urothelial | Pembrolizumab  (270) | Chemotherapy  (272) | 230 | 312 | 0.75  (0.53-1.05) | 0.76  (0.56-1.02) | Yes | Yes | Platinum-based chemotherapy |
| 26412456 | Borghaei et al 2015 | NSCLC | Nivolumab  (292) | Docetaxel  (290) | 339 | 243 | 0.81  (0.62-1.04) | 0.65  (0.44-0.86) | Yes | Yes | Platinum-based chemotherapy |
| 26028407 | Brahmer et al 2015 | NSCLC | Nivolumab  (135) | Docetaxel  (137) | 152 | 120 | 0.52  (0.35-0.75) | 0.59  (0.31-0.87) | Yes | Yes | First-line chemotherapy |
| 28636851 | Carbone et al 2017 | NSCLC | Nivolumab  (271) | Chemotherapy  (270) | 281 | 260 | 1.13  (0.83-1.54) | 1.04  (0.77-1.41) | No | Yes | None |
| 32379280 | Chen et al  2020 | Colorectal | Tremelimumab+  Durvalumab(119) | Best supportive care (248) | 87 | 93 | 0.83  (0.55-1.23) | 0.59  (0.40-0.87) | No | No | All available standard systemic therapies |
| 31003911 | Eng et al  2019 | Colorectal | Atezolozumab+  Cobimetinib(183) | Regorafenib  (90) | 187 | 86 | 0.84  (0.58-1.23) | 1.50  (0.85-2.64) | No | Yes | At least two previous systemic chemotherapy regimens |
| 31003911 | Eng et al  2019 | Colorectal | Atezolozumab  (90) | Regorafenib  (90) | 130 | 50 | 1..20  (0.80-1.81) | 0.99  (0.47-2.07) | NO | Yes | At least two previous systemic chemotherapy regimens |
| 26970723 | Fehrenbacher  et al 2016 | NSCLC | Atezolizumab  (144) | Docetaxel  (143) | 175 | 112 | 0.70  (0.48-1.01) | 0.65  (0.42-0.99) | Yes | No | Platinum-based chemotherapy |
| 29777823 | Fehrenbacher  et al 2018 | NSCLC | Atezolizumab  (425) | Docetaxel  (425) | 453 | 397 | 0.81  (0.65-1.01) | 0.69  (0.54-0.87) | Yes | No | One or two previous cytotoxic chemotherapy regimens |
| 29777823 | Fehrenbacher  et al 2018 | NSCLC | Atezolizumab  (613) | Docetaxel  (612) | 661 | 564 | 0.84  (0.70-1.01) | 0.75  (0.61-0.91) | Yes | Yes | One or two previous cytotoxic chemotherapy regimens |
| 29658856 | Gandhi et al 2018 | NSCLC | Perbrolizumab+  Chemotherapy(410) | Placebo+Chemotherapy(206) | 312 | 304 | 0.43  (0.31-0.61) | 0.64  (0.43-0.95) | No | Yes | None |
| 26712084 | Herbst et al  2016 | NSCLC | Perbrolizumab  (690) | Docetaxel  (343) | 604 | 429 | 0.63  (0.50-0.79) | 0.76  (0.57-1.02) | Yes | Yes | Two or more cycles of platinum-doublet chemotherapy |
| 20525992 | Hodi et al  2010 | Melanoma | Ipilimumab+  gp100(403) | gp100  (136) | 385 | 154 | 0.70  (0.54-0.90) | 0.69  (0.47-1.01) | Yes | Yes | One or two previous systemic treatments |
| 20525992 | Hodi et al  2010 | Melanoma | Ipilimumab  (137) | gp100  (136) | 189 | 84 | 0.65  (0.47-0.90) | 0.61  (0.38-0.99) | Yes | Yes | One or two previous systemic treatments |
| 30280641 | Horn et al  2018 | SCLC | Atezolizumab+  Chemotherapy(201) | Placebo+Chemotherapy(202) | 217 | 186 | 0.92  (0.64-1.32) | 0.53  (0.36-0.77) | No | Yes | None |
| 28993052 | Kang et al  2017 | GEJC | Nivolumab  (330) | Placebo  (163) | 284 | 209 | 0.76  (0.58-1.00) | 0.53  (0.38-0.74) | Yes | Yes | Two or more previous chemotherapy regimens |
| 28671856 | Larkin et al  2018 | Melanoma | Nivolumab  (272) | Investigator's choice(133) | 257 | 148 | 1.17  (0.84-1.63) | 0.62  (0.41-0.94) | Yes | Yes | None |
| 28729154 | Miao et al  2017 | Mesothelioma | Tremelimumab  (382) | Placebo  (189) | 237 | 334 | 0.87  (0.64-1.20) | 0.99  (0.77-1.26) | No | Yes | One or two previous systemic treatments |
| 30955977 | Mok et al  2019 | NSCLC | Pembrolizumab  (637) | Chemotherapy  (637) | 707 | 567 | 0.81  (0.67-0.98) | 0.82  (0.66-1.01) | No | Yes | None |
| 26406148 | Motzer et al 2015 | RCC | Nivolumab  (410) | Everolimus  (411) | 497 | 324 | 0.78  (0.60-1.01) | 0.68  (0.46-0.90) | Yes | Yes | One or two regimens of antiangiogenic therapy |
| 30280635 | Paz-Ares et al 2018 | NSCLC | Perbrolizumab+  Chemotherapy(278) | Placebo+Chemotherapy(281) | 254 | 309 | 0.52  (0.34-0.80) | 0.74  (0.51-1.07) | No | Yes | None |
| 33476593 | Paz-Ares et al 2021 | NSCLC | Nivolumab+Ipilimumab+Chemotherapy(361) | Chemotherapy  (358) | 354 | 295 | 0.61  (0.47-0.80) | 0.62  (0.46-0.85) | No | No | None |
| 30620668 | Reck et al  2019 | NSCLC | Pembrolizumab  (154) | Platinum-based Chemotherapy  (151) | 141 | 164 | 0.60  (0.38-0.96) | 0.64  (0.42-0.98) | No | Yes | None |
| 23295794 | Ribas et al  2013 | Melanoma | Tremelimumab  (328) | Chemotherapy  (327) | 455 | 200 | 0.88  (0.72-1.08) | 0.87  (0.64-1.18) | Yes | Yes | None |
| 30779529 | Rini et al  2019 | RCC | Pembrolizumab+  Axitinib(432) | Sunitinib  (429) | 538 | 323 | 0.47  (0.30-0.73) | 0.59  (0.36-0.97) | No | Yes | None |
| 27979383 | Rittmeyer et al 2016 | NSCLC | Atezolizumab  (425) | Docetaxel  (425) | 453 | 397 | 0.80  (0.64-1.00) | 0.60  (0.52-0.83) | Yes | No | One or more platinum based combination therapies |
| 25399552 | Robert et al  2015 | Melanoma | Nivolumab  (210) | Dacarbazine  (208) | 200 | 218 | 0.52  (0.32-0.85) | 0.33  (0.14-0.52) | Yes | Yes | None |
| 21639810 | Robert et al  2011 | Melanoma | Ipilimumab+  Dacarbazine(250) | Placebo+Dacarbazine(252) | 342 | 160 | 0.44  (0.25-0.74) | 0.81  (0.36-1.78) | No | Yes | None |
| 29880231 | Schitara et al 2018 | GEJC | Pembrolizumab  (196) | Paclitaxel  (199) | 232 | 163 | 0.77  (0.58-1.02) | 0.90  (0.63-1.29) | No | Yes | First-line therapy with platinum, fluoropyrimidine or trastuzumab |
| 30345906 | Schmid et al  2018 | TNBC | Atezolizumab+  Nab-paclitaxel(451) | Placebo+Nab-paclitaxel(451) | 569 | 219 | 0.84  (0.70-1.01) | 0.69  (0.51-0.94) | No | Yes | None |
| 31122901 | West et al  2019 | NSCLC | Atezolizumab+  Chemotherapy(451) | Chemotherapy  (228) | 341 | 338 | 0.79  (0.58-1.08) | 0.78  (0.58-1.05) | No | Yes | None |
| NSCLC, non-small cell lung cancer; RCC, renal cell carcinoma; GEJC, gastric or gastroesophageal junction carcinoma; SCLC, small cell lung cancer; HNC, head and neck cancer; TNBC, triple-negative breast cancer; MESO, mesothelioma. | | | | | | | | | | | |
