# Supplementary material for: Age-Related Differences in Molecular Profiles for Immune Checkpoint Blockade Therapy
Source: Front Immunol. 2021 Apr 15;12:657575. doi: 10.3389/fimmu.2021.657575 (PMC8082107; doi:10.3389/fimmu.2021.657575)
Supplement: Supplementary file 7 [file Table_2.docx]

**Supplementary Table S2.** Molecular biomarkers between the elderly and young patients receiving ICB treatment in clinical trials.

| **Dataset**  **(PMID/Refs)** | **Author**  **Year** | **Cancer**  **type** | **Age (No.)** | | **Drug class** | **Biomarkers** |
| --- | --- | --- | --- | --- | --- | --- |
|  |  |  | **Young**  **(<65)** | **Elderly**  **(≥65)** |  |  |
| 26997480 | Hugo et al  2016 | SKCM | 23 | 15 | anti-PD-1 | TMB, BRCA2 mutation, CTLA4, PD-1, PD-L1, PD-L2, CYT |
| 26359337 | Allen et al  2015 | SKCM | 58 | 47 | anti-CTLA-4 | TMB, neoantigen, BRAF mutation |
| 29657128 | Hellmann et al  2018 | NSCLC | 36 | 36 | anti-PD-1+anti-CTLA-4 | TMB, protein PD-L1, neoantigen |
| 29301960 | Miao et al  2018 | KIRC | 52 | 32 | anti-PD-1 | TMB, PBRM1 mutation |
| 30127394 | Auslander et al 2018 | SKCM | 14 | 23 | anti-PD-1/CTLA-4, Combo | PD-1, PD-L1, PD-L2, CTLA-4, CYT |
| 30309915 | Cristescu et al 2018 | BLCA | 4 | 13 | anti-PD-1 | TMB, GEP |
|  |  | SKCM | 56 | 33 | anti-PD-1 | TMB, GEP |
|  |  | HNSC | 68 | 39 | anti-PD-1 | TMB, GEP |
|  |  | PanCan | 193 | 122 | anti-PD-1 | TMB, GEP |
| 30643254 | Samstein et al 2019 | BLCA | 56 | 83 | anti-PD-1/PDL-1, Combo | TMB |
|  |  | BRCA | 35 | 3 | anti-PD-1/PDL-1, Combo, anti-CTLA | TMB |
|  |  | COAD | 72 | 13 | anti-PD-1/PDL-1, Combo | TMB |
|  |  | ESCA | 63 | 25 | anti-PD-1/PDL-1, Combo | TMB |
|  |  | GBM | 87 | 8 | anti-PD-1/PDL-1, Combo | TMB |
|  |  | HNSC | 63 | 21 | anti-PD-1/PDL-1, Combo | TMB |
|  |  | SKCM | 140 | 96 | anti-PD-1/PDL-1, Combo, anti-CTLA | TMB |
|  |  | NSCLC | 109 | 122 | anti-PD-1/PDL-1, Combo | TMB |
|  |  | KIRC | 82 | 18 | anti-PD-1/PDL-1, Combo | TMB |
|  |  | PanCan | 750 | 413 | anti-PD-1/PDL-1, Combo, anti-CTLA | TMB |
| SKCM, skin cutaneous melanoma; NSCLC, non-small cell lung cancer; KIRC, kidney renal clear cell carcinoma; BLCA, bladder urothelial carcinoma; HNSC, head and neck squamous cell carcinoma; BRCA, breast invasive carcinoma; COAD, colon adenocarcinoma; ESCA, esophageal carcinoma; GBM, glioblastoma; TMB, tumor mutation burden; CYT, cytolytic activity; GEP, T cell-inflamed gene expression profile. | | | | | | |
