# Supplementary material for: Age-Related Differences in Molecular Profiles for Immune Checkpoint Blockade Therapy
Source: Front Immunol. 2021 Apr 15;12:657575. doi: 10.3389/fimmu.2021.657575 (PMC8082107; doi:10.3389/fimmu.2021.657575)
Supplement: Supplementary file 8 [file Table_3.docx]

**Supplementary Table S3.** Summary of sample size for biomarkers analyzed in TCGA dataset.

| **Cancer**  **Type** | **Tumor mutation burden** | | **Immune cell populations, checkpoints, CYT, and GEP** | | **BCR, TCR, neoantigen, and aneuploidy** | | **Protein PD-L1** | |
| --- | --- | --- | --- | --- | --- | --- | --- | --- |
|  | **Young**  **(<65)** | **Elderly**  **(≥65)** | **Young**  **(<65)** | **Elderly**  **(≥65)** | **Young**  **(<65)** | **Elderly**  **(≥65)** | **Young**  **(<65)** | **Elderly**  **(≥65)** |
| BLCA | 120 | 204 | 120 | 204 | 119 | 197 | 99 | 174 |
| BRCA | 562 | 243 | 621 | 273 | 592 | 261 | 507 | 215 |
| CESC | 223 | 27 | 236 | 32 | 226 | 29 | - | - |
| COAD | 92 | 141 | 100 | 150 | 98 | 144 | 68 | 119 |
| ESCA | 81 | 56 | 73 | 47 | 72 | 51 | 52 | 38 |
| GBM | 84 | 56 | 86 | 56 | 86 | 55 | 35 | 24 |
| HNSC | 254 | 165 | 256 | 166 | 259 | 165 | 172 | 109 |
| KIRC | 69 | 70 | 103 | 101 | 95 | 95 | 87 | 84 |
| KIRP | 156 | 106 | 161 | 109 | 156 | 107 | 124 | 77 |
| LAML | 75 | 31 | 107 | 44 | 70 | 28 | - | - |
| LGG | 436 | 33 | 440 | 33 | 441 | 33 | 378 | 23 |
| LIHC | 140 | 133 | 142 | 142 | 135 | 137 | 59 | 80 |
| LUAD | 71 | 82 | 71 | 83 | 50 | 49 | 57 | 56 |
| LUSC | 157 | 284 | 158 | 293 | 152 | 281 | 106 | 188 |
| MESO | 41 | 38 | 45 | 41 | 41 | 39 | 35 | 25 |
| OV | 179 | 95 | 245 | 131 | 232 | 127 | 170 | 90 |
| PAAD | 84 | 68 | 96 | 81 | 88 | 70 | 52 | 46 |
| PCPG | 154 | 24 | 155 | 24 | 138 | 22 | - | - |
| PRAD | 257 | 154 | 263 | 155 | 254 | 145 | 186 | 117 |
| READ | 47 | 40 | 52 | 41 | 52 | 38 | 36 | 34 |
| SARC | 90 | 85 | 96 | 96 | 91 | 89 | 76 | 85 |
| SKCM | 55 | 53 | 55 | 53 | 50 | 53 | 49 | 47 |
| STAD | 146 | 226 | 133 | 205 | 145 | 222 | 123 | 179 |
| THCA | 137 | 61 | 137 | 65 | 132 | 58 | 99 | 52 |
| THYM | 75 | 42 | 76 | 42 | 63 | 39 | 56 | 30 |
| UCEC | 277 | 198 | 286 | 207 | 270 | 197 | 214 | 159 |
| UVM | 45 | 35 | 45 | 35 | 45 | 35 | - | - |
| CYT, cytolytic activity; GEP, T cell-inflamed gene expression profile; BCR, B cell receptor; TCR, T cell receptor; BLCA, bladder urothelial carcinoma; BRCA, breast invasive carcinoma; CESC, cervical squamous cell carcinoma and endocervical adenocarcinoma; COAD, colon adenocarcinoma; ESCA, esophageal carcinoma; GBM, glioblastoma; HNSC, head and neck squamous cell carcinoma; KIRC, kidney renal clear cell carcinoma; KIRP, kidney renal papillary cell carcinoma; LAML, acute myeloid leukemia; LGG, brain lower grade glioma; LIHC, liver hepatocellular carcinoma; LUAD, lung adenocarcinoma; LUSC, lung squamous cell carcinoma; MESO, mesothelioma; OV, ovarian serous cystadenocarcinoma; PAAD, pancreatic adenocarcinoma; PCPG, pheochromocytoma and paraganglioma; PRAD, prostate adenocarcinoma; READ, rectum adenocarcinoma; SARC, sarcoma; SKCM, skin cutaneous melanoma; STAD, stomach adenocarcinoma; THCA, thyroid carcinoma; THYM, thymoma; UCEC, uterine corpus endometrial carcinoma; UVM, uveal melanoma. | | | | | | | | |
